# *N*-(2-Chloro­phen­yl)succinamic acid

**DOI:** 10.1107/S1600536809002979

**Published:** 2009-01-28

**Authors:** B. Thimme Gowda, Sabine Foro, B. S. Saraswathi, Hiromitsu Terao, Hartmut Fuess

**Affiliations:** a Department of Chemistry, Mangalore University, Mangalagangotri 574 199, Mangalore, India; b Institute of Materials Science, Darmstadt University of Technology, Petersenstrasse 23, D-64287 Darmstadt, Germany; c Faculty of Integrated Arts and Sciences, Tokushima University, Minamijosanjima-cho, Tokushima 770-8502, Japan

## Abstract

The conformations of the N—H and C=O bonds in the amide segment of the structure of the title compound {systematic name: 3-[(2-chloro­phen­yl)amino­carbon­yl]propionic acid}, C_10_H_10_ClNO_3_, are *trans* to each other, while the conformation of the amide H atom is *syn* to the *ortho*-chloro group in the benzene ring. Further, the conformations of the amide O atom and the carbonyl O atom of the ester segment are also *trans* to the H atoms attached to the adjacent C atoms. In the crystal structure, mol­ecules are packed into infinite chains through inter­molecular N—H⋯O and O—H⋯O hydrogen bonds.

## Related literature

For general background see: Gowda, Kozisek *et al.* (2007 ▶); Gowda, Svoboda *et al.* (2007 ▶); Gowda *et al.* (2008 ▶); Jones *et al.* (1990 ▶); Wan *et al.* (2006 ▶).
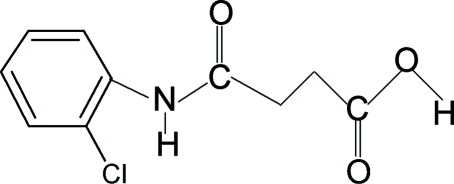


## Experimental

### 

#### Crystal data

C_10_H_10_ClNO_3_
*M* *_r_* = 227.64
Monoclinic, 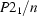

*a* = 4.9056 (5) Å
*b* = 11.126 (1) Å
*c* = 18.677 (2) Å
β = 94.92 (1)°
*V* = 1015.63 (18) Å^3^
*Z* = 4
Mo *K*α radiation
μ = 0.36 mm^−1^
*T* = 299 (2) K
0.50 × 0.35 × 0.30 mm

#### Data collection

Oxford Diffraction Xcalibur diffractometer with a Sapphire CCD detector
Absorption correction: multi-scan (*CrysAlis RED*; Oxford Diffraction, 2007 ▶) *T*_min_ = 0.840, *T*_max_ = 0.899
6644 measured reflections
2065 independent reflections
1585 reflections with *I* > 2σ(*I*)
*R*_int_ = 0.018

#### Refinement

*R*[*F* ^2^ > 2σ(*F* ^2^)] = 0.036
*wR*(*F* ^2^) = 0.126
*S* = 1.08
2065 reflections
142 parameters
2 restraints
H atoms treated by a mixture of independent and constrained refinement
Δρ_max_ = 0.28 e Å^−3^
Δρ_min_ = −0.21 e Å^−3^

### 

Data collection: *CrysAlis CCD* (Oxford Diffraction, 2004 ▶); cell refinement: *CrysAlis RED* (Oxford Diffraction, 2007 ▶); data reduction: *CrysAlis RED*; program(s) used to solve structure: *SHELXS97* (Sheldrick, 2008 ▶); program(s) used to refine structure: *SHELXL97* (Sheldrick, 2008 ▶); molecular graphics: *PLATON* (Spek, 2003 ▶); software used to prepare material for publication: *SHELXL97*.

## Supplementary Material

Crystal structure: contains datablocks I, global. DOI: 10.1107/S1600536809002979/xu2474sup1.cif

Structure factors: contains datablocks I. DOI: 10.1107/S1600536809002979/xu2474Isup2.hkl

Additional supplementary materials:  crystallographic information; 3D view; checkCIF report

## Figures and Tables

**Table 1:** Hydrogen-bond geometry (Å, °)

*D*—H⋯*A*	*D*—H	H⋯*A*	*D*⋯*A*	*D*—H⋯*A*
N1—H1*N*⋯O1^i^	0.877 (16)	2.079 (17)	2.943 (2)	168 (2)
O2—H2*O*⋯O3^ii^	0.814 (18)	1.866 (18)	2.673 (2)	171 (3)

Symmetry codes: (i) 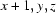; (ii) 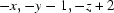.

**Fig. 1.**
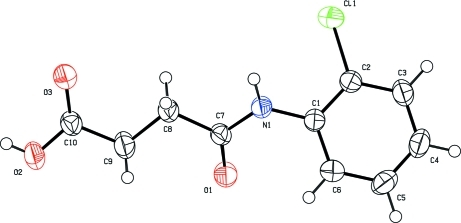
Molecular structure of the title compound, showing the atom labeling scheme. The displacement ellipsoids are drawn at the 50% probability level. The H atoms are represented as small spheres of arbitrary radii.

**Fig. 2.**
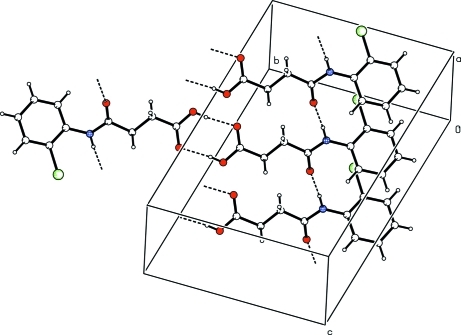
Molecular packing of the title compound with hydrogen bonding shown as dashed lines.

## supplementary crystallographic information

### Comment

Amides are of interest as conjugation between the nitrogen lone pair electrons and the carbonyl pi-bond results in distinct physical and chemical properties. The amide moiety is also an important constituent of many biologically significant compounds. Thus, the structural studies of amides are of interest (Gowda, Kozisek *et al.*, 2007 and references therein; Gowda, Svoboda *et al.*, 2007; Gowda *et al.*, 2008 and references therein); Jones *et al.*, 1990; Wan *et al.*, 2006). As a part of studying the effect of ring and side chain substitutions on the structures of this class of compounds, we have determined the crystal structure of *N*-(2-Chlorophenyl)-succinamic acid (N2CPMSA).

The conformations of N—H and C=O bonds in the amide segment of the structure are *trans* to each other, while the conformation of the amide hydrogen is *syn* to the *ortho*-chloro group in the benzene ring. Further, the conformations of the amide oxygen and the carbonyl oxygen of the ester segment are also *trans* to the H-atoms attached to the adjacent carbons (Fig. 1). The torsional angles of the groups, C1-N1-C7-C8, N1-C7-C8-C9, C7-C8-C9-C10 and C8-C9-C10-O2 in the side chain are 177.5 (2)°, 173.2 (2)°, 178.9 (2)° and 167.7 (2)°, respectively. The molecular packing in the structure *via* N—H···O and O—H···O intermolecular hydrogen bonds (Table 1) is shown in Fig.2.

### Experimental

The solution of succinic anhydride (2.5 g) in toluene (25 ml) was treated dropwise with the solution of 2-chloroaniline (2.5 g) also in toluene (20 ml) with constant stirring. The resulting mixture was stirred for about one hour and set aside for an additional hour at room temperature for completion of the reaction. The mixture was then treated with dilute hydrochloric acid to remove the unreacted 2-chloroaniline. The resultant solid *N*-(2-chlorophenyl)-succinamic acid was filtered under suction and washed thoroughly with water to remove the unreacted succinic anhydride and succinic acid. It was recrystallized to constant melting point from ethanol. The purity of the compound was checked by elemental analysis and characterized by its infrared and NMR spectra. The single crystals used in X-ray diffraction studies were grown in ethanolic solution by slow evaporation at room temperature.

### Refinement

The O-bound and N-bound H atoms were located in difference map, and later restrained to the distance O—H = 0.82 (2) Å, N—H = 0.86 (2) Å, respectivily. The other H atoms were positioned with idealized geometry using a riding model with C—H = 0.93–0.97 Å. All H atoms were refined with isotropic displacement parameters (set to 1.2 times of the *U*_eq_ of the parent atom).

### Crystal data

**Table:**

C_10_H_10_ClNO_3_	*F*(000) = 472
*M**_r_* = 227.64	*D*_x_ = 1.489 Mg m^−^^3^
Monoclinic, *P*2_1_/*n*	Mo *K*α radiation, λ = 0.71073 Å
Hall symbol: -P 2yn	Cell parameters from 2963 reflections
*a* = 4.9056 (5) Å	θ = 2.2–28.0°
*b* = 11.126 (1) Å	µ = 0.36 mm^−^^1^
*c* = 18.677 (2) Å	*T* = 299 K
β = 94.92 (1)°	Rod, colourless
*V* = 1015.63 (18) Å^3^	0.50 × 0.35 × 0.30 mm
*Z* = 4	

### Data collection

**Table:**

Oxford Diffraction Xcalibur diffractometer with a Sapphire CCD detector	2065 independent reflections
Radiation source: fine-focus sealed tube	1585 reflections with *I* > 2σ(*I*)
graphite	*R*_int_ = 0.018
Rotation method data acquisition using ω and φ scans	θ_max_ = 26.4°, θ_min_ = 2.2°
Absorption correction: multi-scan (*CrysAlis RED*; Oxford Diffraction, 2007)	*h* = −6→6
*T*_min_ = 0.840, *T*_max_ = 0.899	*k* = −13→13
6644 measured reflections	*l* = −23→23

### Refinement

**Table:**

Refinement on *F*^2^	Primary atom site location: structure-invariant direct methods
Least-squares matrix: full	Secondary atom site location: difference Fourier map
*R*[*F*^2^ > 2σ(*F*^2^)] = 0.036	Hydrogen site location: inferred from neighbouring sites
*wR*(*F*^2^) = 0.126	H atoms treated by a mixture of independent and constrained refinement
*S* = 1.08	*w* = 1/[σ^2^(*F*_o_^2^) + (0.0713*P*)^2^ + 0.3374*P*] where *P* = (*F*_o_^2^ + 2*F*_c_^2^)/3
2065 reflections	(Δ/σ)_max_ = 0.013
142 parameters	Δρ_max_ = 0.28 e Å^−^^3^
2 restraints	Δρ_min_ = −0.21 e Å^−^^3^

### Special details

**Table:**

Geometry. All e.s.d.'s (except the e.s.d. in the dihedral angle between two l.s. planes) are estimated using the full covariance matrix. The cell e.s.d.'s are taken into account individually in the estimation of e.s.d.'s in distances, angles and torsion angles; correlations between e.s.d.'s in cell parameters are only used when they are defined by crystal symmetry. An approximate (isotropic) treatment of cell e.s.d.'s is used for estimating e.s.d.'s involving l.s. planes.
Refinement. Refinement of *F*^2^ against ALL reflections. The weighted *R*-factor *wR* and goodness of fit *S* are based on *F*^2^, conventional *R*-factors *R* are based on *F*, with *F* set to zero for negative *F*^2^. The threshold expression of *F*^2^ > σ(*F*^2^) is used only for calculating *R*-factors(gt) *etc*. and is not relevant to the choice of reflections for refinement. *R*-factors based on *F*^2^ are statistically about twice as large as those based on *F*, and *R*- factors based on ALL data will be even larger.

### Fractional atomic coordinates and isotropic or equivalent isotropic displacement parameters (Å^2^)

**Table:**

	*x*	*y*	*z*	*U*_iso_*/*U*_eq_	
C1	−0.0798 (3)	0.18091 (17)	0.84736 (10)	0.0321 (4)	
C2	0.0342 (4)	0.22834 (17)	0.78777 (10)	0.0344 (4)	
C3	−0.0244 (4)	0.34392 (19)	0.76422 (12)	0.0443 (5)	
H3	0.0569	0.3749	0.7250	0.053*	
C4	−0.2039 (5)	0.4129 (2)	0.79921 (14)	0.0511 (6)	
H4	−0.2443	0.4907	0.7835	0.061*	
C5	−0.3238 (4)	0.36726 (19)	0.85722 (13)	0.0486 (6)	
H5	−0.4482	0.4137	0.8800	0.058*	
C6	−0.2601 (4)	0.25260 (19)	0.88184 (11)	0.0401 (5)	
H6	−0.3387	0.2232	0.9219	0.048*	
C7	−0.1818 (4)	−0.01912 (17)	0.89519 (10)	0.0337 (4)	
C8	−0.0514 (4)	−0.13863 (18)	0.91553 (12)	0.0421 (5)	
H8A	0.0103	−0.1762	0.8729	0.051*	
H8B	0.1076	−0.1253	0.9491	0.051*	
C9	−0.2446 (4)	−0.2219 (2)	0.94887 (14)	0.0507 (6)	
H9A	−0.4053	−0.2326	0.9154	0.061*	
H9B	−0.3036	−0.1838	0.9916	0.061*	
C10	−0.1304 (4)	−0.34338 (18)	0.96916 (11)	0.0392 (5)	
N1	−0.0075 (3)	0.06395 (15)	0.87205 (9)	0.0369 (4)	
H1N	0.165 (3)	0.042 (2)	0.8730 (12)	0.044*	
O1	−0.4257 (3)	−0.00054 (13)	0.89803 (9)	0.0479 (4)	
O2	−0.2733 (3)	−0.40550 (16)	1.00881 (11)	0.0614 (5)	
H2O	−0.201 (6)	−0.469 (2)	1.0217 (15)	0.074*	
O3	0.0897 (3)	−0.37797 (14)	0.94783 (10)	0.0559 (5)	
Cl1	0.25199 (11)	0.14183 (5)	0.74014 (3)	0.0493 (2)	

### Atomic displacement parameters (Å^2^)

**Table:**

	*U*^11^	*U*^22^	*U*^33^	*U*^12^	*U*^13^	*U*^23^
C1	0.0253 (8)	0.0311 (9)	0.0396 (10)	−0.0008 (7)	0.0003 (7)	0.0032 (8)
C2	0.0285 (9)	0.0339 (10)	0.0410 (10)	−0.0015 (8)	0.0040 (8)	0.0008 (8)
C3	0.0442 (12)	0.0387 (11)	0.0505 (12)	−0.0035 (9)	0.0066 (9)	0.0119 (9)
C4	0.0496 (13)	0.0318 (11)	0.0715 (15)	0.0065 (9)	0.0036 (11)	0.0102 (11)
C5	0.0405 (12)	0.0387 (12)	0.0673 (15)	0.0077 (9)	0.0080 (10)	−0.0066 (10)
C6	0.0366 (10)	0.0419 (11)	0.0426 (10)	0.0020 (9)	0.0088 (8)	0.0008 (9)
C7	0.0285 (9)	0.0357 (10)	0.0374 (10)	0.0009 (8)	0.0047 (7)	0.0075 (8)
C8	0.0321 (10)	0.0372 (11)	0.0583 (13)	0.0034 (8)	0.0105 (9)	0.0143 (10)
C9	0.0361 (11)	0.0423 (12)	0.0748 (15)	0.0018 (9)	0.0106 (10)	0.0231 (11)
C10	0.0317 (10)	0.0382 (11)	0.0477 (11)	−0.0020 (8)	0.0034 (8)	0.0095 (9)
N1	0.0248 (7)	0.0341 (9)	0.0524 (10)	0.0042 (7)	0.0069 (7)	0.0105 (8)
O1	0.0256 (7)	0.0444 (8)	0.0743 (11)	0.0026 (6)	0.0084 (6)	0.0169 (8)
O2	0.0516 (10)	0.0441 (9)	0.0918 (13)	0.0046 (7)	0.0255 (9)	0.0298 (9)
O3	0.0512 (9)	0.0477 (9)	0.0719 (11)	0.0098 (7)	0.0228 (8)	0.0196 (8)
Cl1	0.0475 (3)	0.0468 (3)	0.0566 (4)	0.0016 (2)	0.0224 (2)	−0.0006 (2)

### Geometric parameters (Å, °)

**Table:**

C1—C6	1.390 (3)	C7—N1	1.355 (2)
C1—C2	1.392 (3)	C7—C8	1.510 (3)
C1—N1	1.416 (2)	C8—C9	1.498 (3)
C2—C3	1.381 (3)	C8—H8A	0.9700
C2—Cl1	1.7385 (19)	C8—H8B	0.9700
C3—C4	1.375 (3)	C9—C10	1.500 (3)
C3—H3	0.9300	C9—H9A	0.9700
C4—C5	1.373 (3)	C9—H9B	0.9700
C4—H4	0.9300	C10—O3	1.243 (2)
C5—C6	1.383 (3)	C10—O2	1.267 (2)
C5—H5	0.9300	N1—H1N	0.877 (16)
C6—H6	0.9300	O2—H2O	0.814 (18)
C7—O1	1.220 (2)		
			
C6—C1—C2	117.93 (17)	N1—C7—C8	114.57 (15)
C6—C1—N1	121.89 (17)	C9—C8—C7	112.30 (16)
C2—C1—N1	120.17 (17)	C9—C8—H8A	109.1
C3—C2—C1	121.38 (18)	C7—C8—H8A	109.1
C3—C2—Cl1	118.22 (15)	C9—C8—H8B	109.1
C1—C2—Cl1	120.40 (15)	C7—C8—H8B	109.1
C4—C3—C2	119.5 (2)	H8A—C8—H8B	107.9
C4—C3—H3	120.2	C8—C9—C10	115.23 (17)
C2—C3—H3	120.2	C8—C9—H9A	108.5
C5—C4—C3	120.3 (2)	C10—C9—H9A	108.5
C5—C4—H4	119.9	C8—C9—H9B	108.5
C3—C4—H4	119.9	C10—C9—H9B	108.5
C4—C5—C6	120.23 (19)	H9A—C9—H9B	107.5
C4—C5—H5	119.9	O3—C10—O2	123.9 (2)
C6—C5—H5	119.9	O3—C10—C9	120.93 (18)
C5—C6—C1	120.66 (19)	O2—C10—C9	115.21 (18)
C5—C6—H6	119.7	C7—N1—C1	125.72 (15)
C1—C6—H6	119.7	C7—N1—H1N	115.9 (15)
O1—C7—N1	123.12 (18)	C1—N1—H1N	118.4 (15)
O1—C7—C8	122.29 (17)	C10—O2—H2O	113 (2)
			
C6—C1—C2—C3	1.4 (3)	N1—C1—C6—C5	178.97 (19)
N1—C1—C2—C3	−177.44 (18)	O1—C7—C8—C9	−8.3 (3)
C6—C1—C2—Cl1	−177.74 (14)	N1—C7—C8—C9	173.18 (19)
N1—C1—C2—Cl1	3.5 (3)	C7—C8—C9—C10	178.86 (19)
C1—C2—C3—C4	−1.5 (3)	C8—C9—C10—O3	−12.2 (3)
Cl1—C2—C3—C4	177.60 (17)	C8—C9—C10—O2	167.7 (2)
C2—C3—C4—C5	0.1 (3)	O1—C7—N1—C1	−1.0 (3)
C3—C4—C5—C6	1.4 (4)	C8—C7—N1—C1	177.49 (18)
C4—C5—C6—C1	−1.6 (3)	C6—C1—N1—C7	42.4 (3)
C2—C1—C6—C5	0.2 (3)	C2—C1—N1—C7	−138.8 (2)

### Hydrogen-bond geometry (Å, °)

**Table:**

*D*—H···*A*	*D*—H	H···*A*	*D*···*A*	*D*—H···*A*
N1—H1N···O1^i^	0.88 (2)	2.08 (2)	2.943 (2)	168 (2)
O2—H2O···O3^ii^	0.81 (2)	1.87 (2)	2.673 (2)	171 (3)

Symmetry codes: (i) *x*+1, *y*, *z*; (ii) −*x*, −*y*−1, −*z*+2.
